# Psychometric properties of the Dutch version of the London Measure of Unplanned Pregnancy in women with pregnancies ending in birth

**DOI:** 10.1371/journal.pone.0194033

**Published:** 2018-04-18

**Authors:** Joline Goossens, Sofie Verhaeghe, Ann Van Hecke, Geraldine Barrett, Ilse Delbaere, Dimitri Beeckman

**Affiliations:** 1 University Centre for Nursing & Midwifery, Department of Public Health, Ghent University, Ghent, Belgium; 2 Nursing Science, University Hospital Ghent, Ghent, Belgium; 3 Research Department of Reproductive Health, Institute for Women's Health, University College London, London, United Kingdom; 4 VIVES University College, Kortrijk, Belgium; University of North Carolina at Chapel Hill, UNITED STATES

## Abstract

**Objective:**

To evaluate the psychometric properties of the Dutch version of the London Measure of Unplanned Pregnancy in women with pregnancies ending in birth.

**Methods:**

A two-phase psychometric evaluation design was set-up. Phase I comprised the translation from English into Dutch and pretesting with 6 women using cognitive interviews. In phase II, the reliability and validity of the Dutch version of the LMUP was assessed in 517 women giving birth recently. Reliability (internal consistency) was assessed using Cronbach’s alpha, inter-item correlations, and corrected item-total correlations. Construct validity was assessed using principal components analysis and hypothesis testing. Exploratory Mokken scale analysis was carried out.

**Results:**

517 women aged 15–45 completed the Dutch version of the LMUP. Reliability testing showed acceptable internal consistency (alpha = 0.74, positive inter-item correlations between all items, all corrected item-total correlations >0.20). Validity testing confirmed the unidimensional structure of the scale and all hypotheses were confirmed. The overall Loevinger’s H coefficient was 0.57, representing a ‘strong’ scale.

**Conclusion:**

The Dutch version of the LMUP is a reliable and valid measure that can be used in the Dutch-speaking population in Belgium to assess pregnancy planning. Future research is necessary to assess the stability of the Dutch version of the LMUP, and to evaluate its psychometric properties in women with abortions.

## Introduction

Unplanned pregnancies have been associated with more unhealthy perinatal behavior and an increased risk of several adverse antenatal and birth outcomes, including spontaneous abortion, congenital anomalies, preterm birth, and low birth weight [1–6]. Prevention of unplanned pregnancy has become a global health priority [7–10].

Estimating the prevalence of unplanned pregnancy is important for the design and evaluation of effective preconception care initiatives and strategies for unintended pregnancy prevention, for example school- or community-based sex education programs, contraceptive education programs, and the provision of no-cost (emergency) contraception [11–14]. In the past, there have been numerous attempts to measure pregnancy planning, primarily by the means of survey questions. Many of these studies fail to measure pregnancy planning adequately due to methodological challenges, including lack of clear definitions, the utilization of measures without rigorous psychometric evaluation, and difficulties with conceptualizing pregnancy intention or planning [15–17]. Most conventional measures of pregnancy intention or planning assume that becoming pregnant is a conscious choice and/or women have well defined family building plans [15, 18–20]. For example, the 2013–2015 National Health and Family Growth (NSFG)–the primary data source on pregnancy intention in the United States–asks a series of questions regarding the timing and desire for children including; ‘Right before you became pregnant …, did you yourself want to have a(nother) baby at any time in the future?’[21]. The pregnancy intendedness is categorized into three categories: ‘intended’, ‘mistimed’, and ‘unwanted’. A pregnancy is categorized as ‘intended’ if a woman indicated that her pregnancy occurred at the time she wanted to become pregnant, or later, or if she didn’t care about the timing of the pregnancy. A pregnancy is classified as ‘mistimed’ if the pregnancy occurred sooner than the woman wanted. If a woman states she wanted no more children for the rest of her life, it was categorized as ‘unwanted’. ‘Unintended’ pregnancies refer to ‘mistimed’ and ‘unwanted’ pregnancies [22]. The problem with measures that categorize pregnancy intention and planning in a dichotomous manner, namely intended *versus* unintended and planned *versus* unplanned, is that it leads to oversimplification of a complex construct [17, 18]. Several studies have shown that some women experience conflicting attitudes and feelings towards preventing a pregnancy or fail to form explicit intentions about their fertility, which can result in inadequate contraceptive use [19, 23–26]. This complexity is rarely captured in conventional survey questions, and therefore, continuous or multi-item measures might be more appropriate to measure the construct of pregnancy intention or planning [17, 18, 27, 28]

The London Measure of Unplanned Pregnancy (LMUP) is an instrument that takes the complexity of pregnancy planning into account [29]. The LMUP was developed in the United Kingdom (UK) based on qualitative research, and has been assessed as valid and reliable (Cronbach’s α = 0.92, test–retest = 0.97) [29]. It does not assume that women have fully developed childbearing plans nor that their behavior is consistent with their intentions, which allows them to express ambivalence about becoming pregnant. Because previously conducted qualitative research suggests that women do not differentiate between the terms ‘planning’ and ‘intending’ a pregnancy, these terms are used as synonyms [19, 30]. The LMUP is a short, inoffensive, easy to understand, self-administered measure, and therefore, suitable for use in large scale studies (original version in English is available at www.lmup.org.uk). The LMUP has been translated into other languages including Spanish, Portuguese, Urdu, Arabic, and Persian; and its psychometric properties have been evaluated in different populations and settings [30–36]. The LMUP may also be a useful tool for assessing the prevalence of unplanned pregnancies in Dutch-speaking regions as no national data registration or questionnaires are available. However, psychometric properties of the Dutch translation of the LMUP are required. Therefore, the aim of this analysis, as part of the wider study on pregnancy planning in Flanders [4], was to translate the LMUP into Dutch and to evaluate its psychometric properties (validity and reliability) among women in Flanders, Belgium, who had a pregnancy ending in birth.

### Study context

Belgium is a federal, high-income country in North-Europe with a population of 11 million people. The two largest regions in the country are the Dutch-speaking region of Flanders in the north and the French-speaking region in the south (Wallonia). The Brussels-Capital region is an officially bilingual (French and Dutch) [37]. Approximately 25% of the Belgian population are women of reproductive age (15–45 years) [38]. Over the past few decades, the fertility of women in Flanders has followed a trend toward postponement and decline of childbearing. In 1991, the mean age at first motherhood was 26.3 years, and steadily increased over the years to a mean age of 28.8 years in 2015 [39]. In 2008, the total fertility rate (TFR) in Flanders was estimated at 1.66 children per woman; 1.77 in 2012; and further dropped to 1.66 in 2015 [40]. Women in Flanders have easy access to birth control and emergency contraception. Data from the Belgian National Health Interview Survey of 2013 showed that 74% of the Flemish sexually active women aged 15–54 (or their partner) used contraception, including emergency contraction [41, 42]. Official statistics showed that Belgium has one of the lowest abortion rates of the world, 9 abortions per 1,000 reproductive-age women [43]. Belgian women who meet legal requirements (up to 12 weeks of gestation) have easy access to abortion services.

## Methods

The study included two phases. Phase I comprised the translation and adaption of the LMUP into the Dutch language. Phase II assessed the reliability and validity of the Dutch version of the LMUP.

### Instrument

The LMUP measures retrospectively the extent to which the most recent pregnancy was planned/intended through six items related to stance (intention to conceive, and desire for a baby), context (timing of motherhood, and discussion with partner), and behavior (contraceptive use, and preconceptional preparations). Each item is scored zero, one or two, with a total sum score from zero to 12. Total scores can be treated as continuous, with a higher score indicating a higher degree of pregnancy planning [29, 44]. The total score can also be divided into three groups: 0–3 (‘unplanned’), 4–9 (‘ambivalent’), and 10–12 (‘planned’), or dichotomized at scores 9/10 (unplanned/planned), depending on the requirements of the analysis [29, 44].

### Phase I: The Dutch translation and adaption of the LMUP

The LMUP was translated, guided by the World Health Organization (WHO)’s process of translation and adaption of research instruments that consists of four steps: (1) forward translation, (2) expert panel, (3) backward translation, and (4) pre-testing and cognitive interviewing with a minimum of 10 respondents [45]. Due to time constraints, not all steps of this process were fully followed. First, two Dutch-speaking researchers (JG and SDB) who were fluent in English independently translated the LMUP into Dutch. A back-translation was not performed, but instead a third Dutch-speaking researcher (AVL) checked the adequacy of the translation, as well as the comprehensibility of the items. Next, the few discrepancies between the forward translation and the original wording, and suggestions for alternative expressions were discussed between the researchers. Finally, the translated questionnaire was pre-tested using cognitive interviewing techniques. Because of time constraints and the fact no new information was obtained in the last interviews, a sample of only 6 women who met the inclusion criteria were included in the pre-test. Women were recruited by snowball sampling, and were interviewed at home by a trained interviewer (JG). Respondents were instructed to read each item and response option aloud, and to explain what the question is asking, and whether they could rephrase the question in their own words. Respondents were also asked about wording they did not understand, as well as any words or expression that they found offensive or unacceptable. The women in the pre-test had mean age of 28.5 years with a range of 22 to 31 years. Most of them were low (n = 1) or medium educated (n = 3), and two women had college or university education. No immigrant women were interviewed.

Based on the debriefing of the pre-test respondents, minor changes were made. Firstly, one woman stated she did not understand the term “contraception” in the response options of item one, therefore, the term was replaced by “birth control”. Secondly, three preconception health behaviors were added to the list of response options of item six: “I used multivitamins”, “I stopped or reduced the consumption of caffeine containing drinks”, and “I achieved a healthier weight”. These actions were added because they are more commonly taken by Flemish women preparing for pregnancy (multivitamin use), and because of international recommendations and studies on preconception health (reduction of caffeine and achieving a healthy weight) [46–48]. Adaptations to item 6 to ensure its local relevance is well established [30, 34] and supported by the developer (GB). Finally, three examples of commonly consumed caffeine containing drinks were added in parentheses (coffee, tea, cola soda,…) because two women reported they were unsure which drinks contain caffeine (S1 Instrument).

### Phase 2: Reliability and validity of the LMUP

#### Participants and procedures

A detailed description of the methodology of this study is described elsewhere [4]. Briefly, 517 women (22% response rate) were enrolled in the study between March through September 2015 via six non-teaching public Hospitals in Flanders (Belgium). Women were eligible to participate if they were 1) admitted to the postnatal maternity ward, 2) between 15 and 45 years old, and 3) Dutch speaking. The head or study midwife of each postnatal ward was asked to approach all eligible women, and to inform them about the aim and procedures of the study on preparations before pregnancy. Women who agreed to participate in the study completed a one-time questionnaire during the first five days postpartum, and returned it in a sealed envelope to a midwife. Additionally, information on pregnancy, delivery, and birth outcomes was collected from medical records by two junior researchers (qualified midwives an master students in Midwifery). All the data were anonymised before further processing to ensure confidentiality. The study was approved by the Ethics Committee of Ghent University Hospital (B670201524084 & B670201524085) and all six local research ethics committees. All participants provided written informed consent. For women under the age of 18 years, written informed consent was also obtained from parents or legal guardians.

#### Data analysis

Psychometric properties of the Dutch version of the LMUP were assessed using the Classical Test Theory-based (CTT) approach to facilitate comparison with the original UK study [29] and previous validations [30–36]. Data were analyzed using Statistical Package for the Social Sciences (SPSS) version 21 (IBM Corporation, Armonk, NY, USA) and Stata version 13 (Stata Corporation, College Station, TX, USA). P-values ≤ 0.05 were considered statistically significant.

Performance of the LMUP items: The amount of missing data for each item was assessed. Total scores were obtained by summing the score on each item; the three added actions of item six were assessed as “taking another action”. For participants missing one to three of the six item responses, scores for the missing items were imputed by using a mean score of the non-missing items allowing a total score to be calculated. If more than three items were missing, imputation of missing data was not carried out and no total score was calculated [29]. Item endorsement frequencies were calculated for each item to investigate if there were response options with a very high (> 80%) selection.

An analysis of the distribution of the total scores was performed to ensure the full range of scores were present and to evaluate the targeting of the measure. The readability of the Dutch version of the LMUP was evaluated by using the Flesch Reading Ease Score (FRES; 100-point scale; the higher the score, the easier to understand) and Flesch-Kincaid Grade Level (FKGL)[49].

Reliability of the LMUP: The internal consistency was evaluated by calculating the Cronbach’s alpha coefficient, using 0.7 as cutoff for acceptable reliability [50]. In addition, all corrected item-total correlations were assessed, with scores above 0.2 indicating an acceptable correlation between each item and the overall score [51]. Moreover, inter-item correlations were calculated to verify that all items were positively correlated.

Validity of the LMUP: Construct validity was assessed by using principal components analysis and hypothesis testing. The principal components analysis (PCA) was used to determine the number of underlying constructs. The LMUP was considered as valid if all items loaded onto one construct with an Eigenvalue greater than one [50]. Construct validity hypotheses, using the known groups technique, were formulated a priori and were based on the findings from the original UK study [29] and from literature [3, 52–57]. We hypothesized that the following women would have a lower level of pregnancy planning, and thus, lower LMUP median scores: 1) single women and women living without their partner, 2) women with lower educational attainment, 3) immigrant women, 4) younger women, 5) multiparous women, 6) women having difficulty making ends meet, 7) women without a paid employment, 8) those experiencing intimate partner violence Level of education was recoded as “low” (primary, secondary or post-secondary education) and “high” (college or university education). Ethnicity was based on country of birth of the parents, and a woman was classified as “immigrant” if one of her parents was born outside Belgium. Subjective poverty was based on the European Union–Statistics on Income and Living Conditions (EU-SILC), and was measured by asking, “How easy or difficult is it to make ends meet?” (easy, rather easy, rather difficult, difficult) [58]. For analyses, responses were recoded to easy/rather easy and difficult/rather difficult. Participants with a partner were asked to complete three items assessing physical, emotional, and sexual Intimate Partner Violence (IPV) that were based on a study by Galle and colleagues [59]. Hypotheses were tested using the non-parametric Mann-Whitney U or Kruskal–Wallis test.

Exploratory Mokken analysis: In keeping with several previous evaluations of the LMUP [31, 33], a Mokken scale analysis was conducted. Mokken scaling is a non-parametric method derived from Item Response Theory (IRT) and is a probabilistic version of Guttman scaling [60, 61]. Mokken’s model assumes the existence of an underlying construct (in this study pregnancy planning) which is captured by a homogenous set of items. Items vary in difficulty, and are hierarchically ordered by their degree of difficulty: people endorse an ‘easier’ item before they endorse a ‘harder’ or less popular item [60, 61]. Loevinger’s coefficient (H) is a parameter of the scalability, which is the extent to which items will be ordered hierarchically relative to one other based on their mean values. The fewer violations of Guttman ordering, the greater the scalability and the higher the H values [60]. Items with a H value > 0.3 were eligible for scaling [62, 63]. The scalability of the full scale was also assessed, with H values < 0.4 indicating a 'weak' scale, 0.40–0.49 a ‘medium’ scale, and ≥ 0.50 a ‘strong’ scale [63].

## Results

The socio-demographic characteristics of the 517 women in the sample are described in Table 1. The majority of the reproductive-aged women were multiparous, in a relationship, with a high educational background.

**Table 1:** Socio-demographic characteristics of the study participants (n = 517).

Characteristic	M	SD
Age (years)	29.5	0.2
**Characteristic**	**n**	**%**
Gravida		
First pregnancy	201	39.0
Second or subsequent pregnancies	314	61.0
Parity		
First birth	247	48.0
Second or subsequent births	268	52.0
Nationality		
Belgian nationality	494	95.7
Other nationality	22	4.3
Ethnicity^a^		
Natives	455	88.5
Immigrants	59	11.5
Education^b^		
Low	19	3.7
Medium	181	35.3
High	313	61.0
Paid employment		
No	55	10.7
Yes	457	89.3
Monthly net household income		
< €2.000	44	8.8
€2.000 - €3.000	98	19.7
> €3.000	356	71.5
Subjective poverty		
Making ends meet with difficulty	74	14.7
Making ends meet easily	428	85.3
Partnership status		
Cohabiting with husband/partner	498	96.5
Not cohabiting with husband/partner	11	2.1
No current partner	7	1.4
Intimate partner violence (IPV)^c^	18	3.5

^a^One of the parents born outside

^b^Level of education: low = primary education, medium = secondary or post-secondary education, high = college or university education.

^c^Physical, emotional or sexual IPV.

Abbreviations: M, mean; SD, standard deviation.

### Performance of the LMUP items

In general, the number of missing responses was very low (0.2%– 0.6%). Three participants (0.6%) had missing responses on one item, and one participant failed to respond to two items (0.2%). One participant had missing responses on four items (0.2%), and therefore, total LMUP score could not be calculated. Items with the most missing responses were item 5 (partner discussion) and item 1 (contraception)(Table 2).

**Table 2:** Endorsement frequencies of LMUP items and response options.

Item	Category	N	%
1. Contraception	0. Always using contraception	11	2.1
	1. Using sometimes or failed at least once	24	4.6
	2. Not using contraception	480	92.8
	Missing data	2	0.4
2. Timing	0. Did not want pregnancy at all	6	1.2
	1. Wanted pregnancy later	38	7.4
	2. Wanted pregnancy then or sooner	472	91.3
	Missing data	1	0.2
3. Intention	0. Did not intend pregnancy	37	7.2
	1. Intentions kept changing	20	3.9
	2. intended pregnancy	459	88.8
	Missing data	1	0.2
4. Desire	0. Did not want baby	9	1.7
	1. Mixed feelings about having baby	39	7.5
	2. Wanted baby	468	90.5
	Missing data	1	0.2
5. Partner	0. Never discussed getting pregnant	7	1.4
	1. Discussed but did not agreed to get pregnant	39	7.5
	2. Agreed to get pregnant	468	90.5
	Missing data	3	0.6
6. Preparation	0. Did no preparatory lifestyle changes	134	25.9
	1. Did 1 preparatory lifestyle change	155	30.0
	2. Did 2 or more preparatory lifestyle changes	184	43.9
	Missing data	1	0.2

Five items (contraception, timing, intention, desire, partner) had a response option with more than 80% endorsement: over 80% of the respondents did not use contraception when they became pregnant (item 1, category 2), wanted to become pregnant then or sooner (item 2, category 2), intended to become pregnant (item 3, category 2), wanted to have a baby when they became pregnant (item 4, category 2), and agreed with their partner to get pregnant (item 5, category 2). Few participants selected the first of the response options on these five items (Table 2).

**Fig 1:**
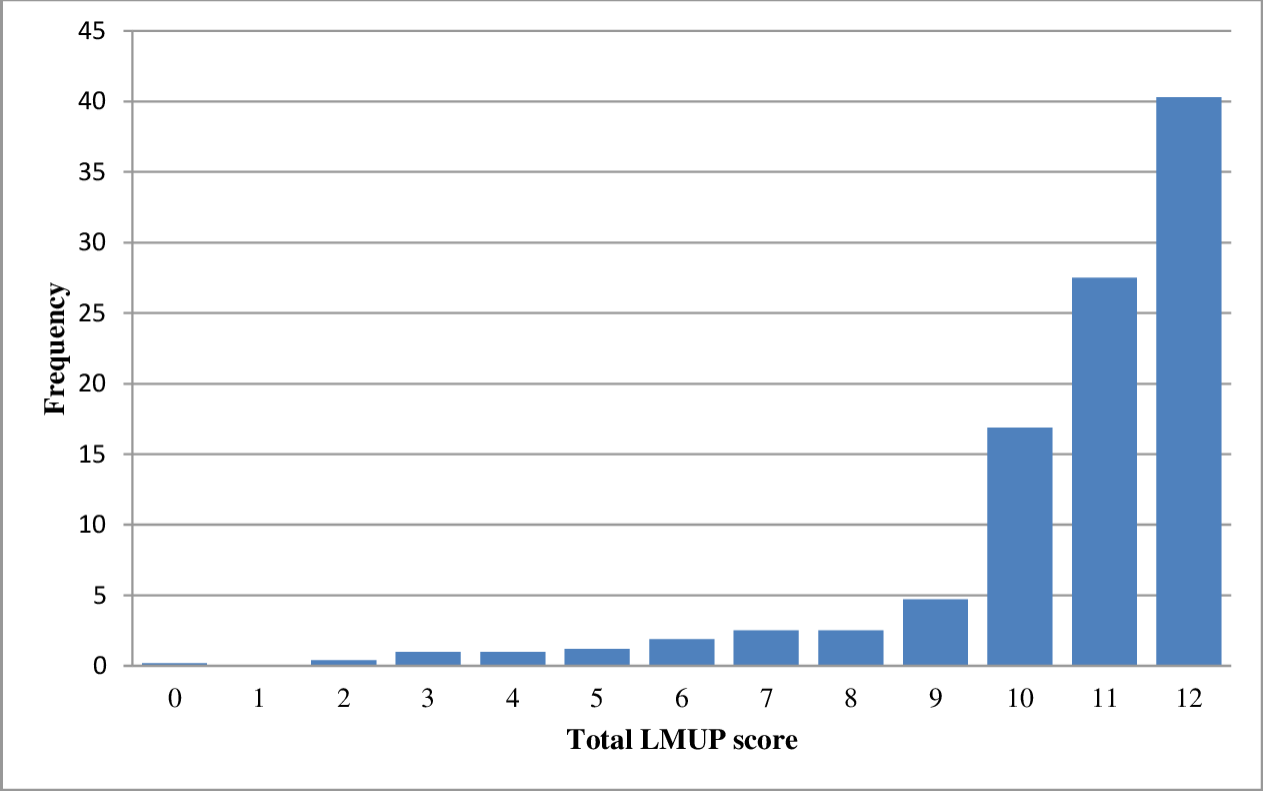
Distribution of Dutch London Measure of Unplanned Pregnancy (LMUP) scores.

The distribution of the total LMUP scores was strongly left-skewed (Fig 1). The median score was 11 (inter-quartile range 10–12), with 437 (84.7%) participants scoring 10–12 (planned); 71 (13.8%) scoring 4–9 (ambivalent); and 8 (1.6%) scoring 0–3 (unplanned).

The Dutch version of the LMUP scored 7.4 on the Flesch-Kincaid Grade Level score and rated 73% on the Flesch Reading Ease score, which both corresponds to a 7^th^ grade reading level or a reading age of 12 years.

### Reliability of the LMUP

Alpha was 0.74 and all corrected item-total correlations were above 0.20 (item 1: 0.40, item 2: 0.57, item 3: 0.69, item 4: 0.59, item 5: 0.69, item 6: 0.32). All inter-item correlations were in the positive direction, and showed moderate and strong correlations between the items (range: 0.14–0.69).

### Validity of the LMUP

Principal component analysis confirmed the one-dimensional structure of the scale (Eigenvalue = 3.17), with factor loadings above 0.45 on all items (item 1: 0.58, item 2: 0.76, item 3: 0.86, item 4: 0.78, item 5: 0.84, item 6: 0.45). All hypotheses were confirmed (Table 3).

**Table 3:** Construct validity hypothesis tests.

Hypothesis	Variable	Score range (median)	P-value
Living with partner will be associated with higher scores, other categories with lower scores.	Partnership status:		
	Cohabiting with husband/partner	2–12 (11)	
	Not cohabiting with husband/partner	2–12 (9)	0.001^a^
	No current partner	0–12 (8)	
Higher educational status will be associated with higher scores.	Educational level:		
	None	4–11 (10)	< 0.001^a^
	Primary	7–12 (11)	
	Secondary	2–12 (11)	
	Post-secondary	0–12 (11)	
	College or university	3–12 (11)	
Natives will have the highest scores.	Ethnicity		
	Natives	0–12 (11)	0.05 ^b^
	Immigrants	3–12 (11)	
The youngest women will have the lowest scores.	Age group		
	< 20	7–9 (8)	0.005^a^
	20–24	0–12 (11)	
	25–29	5–12 (11)	
	30–34	2–12 (11)	
	35–39	3–12 (11)	
	40+	8–12 (12)	
Nulliparous women will have the highest scores.	Number of children		
	First child	2–12 (11)	<0.001^a^
	Second child	3–12 (10)	
	Third or more child	3–12 (10)	
Making ends meet easily will have the highest scores.	Subjective poverty		
	Making ends meet with difficulty	2–12 (10)	<0.001^b^
	Making ends meet easily	0–12 (11)	
Paid employment will be associated with highest scores.	Paid employment		
	Yes	0–12 (11)	0.001^b^
	No	2–12 (11)	
Intimate partner violence will be associated with lowest scores.	Intimate partner violence		
	Yes	0–12 (7.5)	<0.001^b^
	No	2–12 (11)	

^a^Kruskal-Wallis tests

^b^Mann-Withney U tests

### Exploratory Mokken analysis

The Mokken scale analysis showed an hierarchical ordering of the items according to their difficulty, with item 2 (timing of motherhood) being the easiest to endorse, followed by items 5, 4, 1, 3, and 6 (preconceptual preparations) being the most difficult to endorse. All items were eligible for scaling (H > 0.3) and successfully formed a Guttman scale (H: item 1, 0.42; item 2, 0.54; item 3, 0.64; item 4: 0.55; item 5: 0.63; item 6: 0.58). The overall Loevinger’s coefficient was 0.57, representing a ‘strong’ scale.

## Discussion

The objective of this study was to translate the London Measure of Unplanned Pregnancy (LMUP) into Dutch and to evaluate its psychometric properties in a sample of Dutch-speaking women giving birth recently.

Like the original LMUP [29] and the other translated versions [30, 31, 33, 34, 36], the Dutch version of the LMUP items had very low rates of missing data. The readability level was at 7^th^ grade level, which is again in line with the original LMUP (6^th^– 7^th^ grade) [29] and the US English version (6^th^ grade) [31]. By contrast, the Dutch version of the LMUP performed less well on the 80% endorsement criterion which gives an indication of item discrimination. The first of the three response options of item one to five (contraception, timing, intention, desire, partner) received very high response rates (89–93%). This can, however, be easily explained by the fact that our study population was homogeneous in terms of pregnancy outcome–that is, pregnancies ending in birth. It is not surprising that in a high-income country with easy access to (emergency) birth control and legal abortion, and a study population consisting of women with a continuing pregnancy, the proportion of planned pregnancies is higher compared to studies in low-income countries, countries with difficult access to (emergency) birth control or where abortion is illegal, and studies that included the abortion population. This also explains the high response rates to categories corresponding with a higher degree of pregnancy planning, and the reason why the distribution of the total LMUP scores was strongly left-skewed. It is surprising that item 6 did not show the same endorsement pattern as item one to five, i.e. a high proportion of women reporting two or more preconception lifestyle changes. However, similar as described in the Brazilian and Iranian study [32, 33], women in Belgium are not familiar with the concept of preconception health and care. To date, there is little experience with implementing preconception care initiatives in the Dutch-speaking part of Belgium. While this study on pregnancy planning was conducted, an evidence-based website on preconception care with a specific focus on folic acid intake was launched evidence-based information for both women and men planning a pregnancy, and healthcare providers [64]. It is possible that the implementation of this website and other future preconception initiatives, will lead to making more preconception lifestyle changes, and thus, higher responses to categories 2 and 3 of item 6.

Our findings support the reliability of the Dutch version of the LMUP. The internal consistency was acceptable with a Cronbach's alpha of 0.74, comparable to the versions in India, Malawi, and the US (α = 0.71–0.78) [30, 31, 34], but lower than the original English measure (α = 0.92) [29] and the Portuguese, Persian, Urdu, Arabic, and Spanish versions of the LMUP (α = 0.81–0.87) [31–33, 35, 36].

Our results of the principal component analysis confirmed the unidimensionality of the LMUP, which provides evidence for the construct validity of the Dutch version of the LMUP. These findings are consistent with those reported from the original UK study [29], and from Brazil [33], Pakistan [36], Saudi Arabia [35], and the United States [31]. In addition, all hypotheses were confirmed, providing further support for the construct validity of the Dutch version of the LMUP.

The results of the Mokken analysis also confirmed that all six items contributed to the scale, which is consistent with findings of the Brazilian study [33]. In comparison, the Mokken analysis of the US version of the LMUP indicated that the contraception item contributed only little to the scale [31]. The US study was conducted in low-income women with a limited access to birth control. In contrast, Belgian women have an easy access to contraception [41], which might explain this difference.

This study has some limitations. First, due to time constraints, the WHO’s process of translation and adaption of research instruments was not fully followed. There was no back-translation and external expert panel, and the pre-test consisted of only six, native women instead of the recommended minimum of 10. However, the women in the pre-test sample were from different age and socioeconomic groups, and during the last interviews no new information was obtained. In addition, the WHO guidelines are more stringent compared to other guidelines, such as the COSMIN standards for cross-cultural validity of a measure, of which most standards are met in this study [65]. Another important limitation of our study is the lack of test-retest reliability data for the Dutch version of the LMUP. In addition, we did not include women with a pregnancy ending in abortion, which resulted in a homogeneous sample. Thus, the psychometric properties of the LMUP are unknown in the abortion population, and it is therefore important to confirm that the Dutch version of the LMUP is also valid for use among women with a pregnancy ending in abortion. Finally, higher educated and native-born women were overrepresented in our study compared to the general population of women giving birth in Flanders [66]. On the other hand, this study has several strengths, including a rigorous process of translation and adaption of the LMUP from English to Dutch, a large sample size, and a comprehensive database on several aspects of the pregnancy enabling hypothesis-testing. This was the first validation study that examined large number of hypotheses based on findings from the original UK study and existing literature to support the construct validity of the LMUP.

Future validation studies should include women during the first trimester of their pregnancy in order to include pregnancies ending in birth, as well as induced and spontaneous abortions. In addition, the test-retest reliability should be examined. The longer term stability of women’s reported intentions, for instance between pregnancy and after birth, may also be assessed in future.

## Conclusion

This study supports the reliability and validity of the Dutch version of the Belgian LMUP. The Dutch version of the LMUP measure can be used to study unplanned pregnancies in the Dutch-speaking population in Belgium as public health research on this topic is lacking in in Belgium. It would also be interesting to use the LMUP in intervention development and evaluation regarding preconception care and the reduction of unplanned pregnancies [14]. Before an intervention can be developed, it is necessary to conduct a needs assessment and a problem analysis to understand the problem and to identify what needs to be changed [67, 68]. For example, the LMUP can be used to gain insight in the prevalence of planned and unplanned pregnancies, the associated factors and underlying processes, and the maternal and neonatal outcomes. These insights can contribute to the development of an intervention to increase the number of well-planned pregnancies. Future research, however, is necessary to assess the stability of the Dutch version of the LMUP, and to evaluate its psychometric properties in women with abortions.

## Supporting information

S1 Instrument The Dutch version of the London Measure of Unplanned Pregnancy. (DOCX)
