# Supplementary material for: Psychometric properties of the Dutch version of the London Measure of Unplanned Pregnancy in women with pregnancies ending in birth
Source: PLoS One. 2018 Apr 18;13(4):e0194033. doi: 10.1371/journal.pone.0194033 (PMC5905964; doi:10.1371/journal.pone.0194033)
Supplement: S1 Instrument — (DOCX) [file pone.0194033.s001.docx]

**S1 Instrument. The Dutch version of the London Measure of Unplanned Pregnancy.**

Hieronder vind je enkele vragen die peilen naar je situatie en gevoelens in de periode waarin je zwanger werd. Denk aan jouw meest recente zwangerschap bij het beantwoorden van de vragen.

1. **In de maand dat ik zwanger werd…** *(duid aan welke stelling het best bij je past):*

- Gebruikte ik/wij geen voorbehoedsmiddelen
- Gebruikte ik/wij voorbehoedsmiddelen, maar niet altijd
- Gebruikte ik/wij altijd voorbehoedsmiddelen, maar weet ik dat de methode minstens eenmaal heeft gefaald (vb. verschoven, werkte niet, ...)
- Gebruikte ik/wij altijd voorbehoedsmiddelen

1. **In termen van ‘moeder worden’ (eerste keer of opnieuw), heb ik het gevoel dat mijn zwangerschap plaatsvond op een…** *(duid aan welke stelling het best bij je past):*

- Juist moment
- Ok, maar niet echt het juist moment
- Verkeerd moment

1. **Net vóór ik zwanger werd…** *(duid aan welke stelling het best bij je past):*

- Was ik van plan om zwanger te worden
- Veranderden mijn plannen om zwanger te worden voortdurend
- Was ik niet van plan om zwanger te worden

1. **Net vóór ik zwanger werd…** *(duid aan welke stelling het best bij je past):*

- Wilde ik een baby krijgen
- Had ik gemengde gevoelens over het krijgen van een baby
- Wilde ik geen baby krijgen

*De volgende vraag heeft betrekking op je partner – dit is (of was) mogelijks je echtgenoot, een partner waarmee je samenleeft, een vriendje, of iemand waar je één of twee keer seksuele betrekking mee had.*

1. **Vóór ik zwanger werd…** *(duid aan welke stelling het best bij je past):*

- Waren mijn partner en ik het erover eens dat we zwanger wilden worden
- Hadden mijn partner en ik het samen krijgen van kinderen besproken, maar waren we niet overeengekomen om zwanger te worden
- Hadden mijn partner en ik het samen krijgen van kinderen nooit besproken

1. **Vóór je zwanger werd, heb je iets gedaan om je gezondheid te bevorderen als voorbereiding op de zwangerschap?** (*Er zijn meerdere antwoorden mogelijk)*

- Ik nam foliumzuur
- Ik ben gestopt of verminderd met roken
- Ik ben gestopt of verminderd met het drinken van alcohol
- Ik at gezonder
- Ik zocht medisch/gezondheid advies
- Ik ondernam een andere actie, gelieve te omschrijven:………………………………

of

- Ik ondernam niets van bovenstaande acties vóór mijn zwangerschap
